# Sub specialization and centralization of pediatric neuro-oncology services: a strategy to improve outcomes in low-resource settings

**DOI:** 10.3389/fonc.2025.1618469

**Published:** 2025-12-12

**Authors:** Abigail Baker, Vasudeva Bhat K., Daniel C. Moreira, Ibrahim Qaddoumi

**Affiliations:** 1 College of Natural and Health Sciences, Southeastern University, Lakeland, FL, United States; 2 Department of Pediatric Oncology, Kasturba Medical College, Manipal, Manipal Academy of Higher Education, Manipal, Karnataka, India; 3 Department of Global Pediatric Medicine, St. Jude Children’s Research Hospital, Memphis, TN, United States

**Keywords:** pediatric neuro-oncology, subspecialization, centralization, LMIC (low and middle income countries), neuro oncology

## Abstract

**Background:**

Subspecialization and centralization increase the quality of care in many medical subspecialties. It is expected to be standard of care in high-income countries. However, studies addressing the effect of subspecialization and centralization on the quality of pediatric neuro-oncology care in low-resource settings are lacking.

**Methods:**

We determined the impact of subspecialization and centralization of pediatric neuro-oncology services on quality of care, survival outcomes, and quality of life by searching the Medline/PubMed literature database to identify relevant articles. We conducted this search using multiple English terms and keywords.

**Results:**

Pediatric brain tumors are rare, and the care of pediatric neuro-oncology patients is complex and requires a multidisciplinary team. Established pediatric neuro-oncology programs greatly increase patient survival, outcomes, quality of care, and hospital volume. Barriers to centralization may include the distance to travel and the financial burden of that travel, which could lead to underserved populations.

**Conclusions:**

Subspecialization and centralization of pediatric neuro-oncology services improves quality of care and patient outcome. More prospective research in this area is needed to determine the true impact of subspecialization and centralization in pediatric neuro-oncology in low-resource settings.

## 1 Introduction

Survival of children with cancer continues to improve as more advanced, effective therapies are developed. However, this improvement is mostly benefiting those children in high-income countries (HICs) (1). Where a child is born and resides is still a major predictive measure of survival (2). More than 211,080 children receive a cancer diagnosis each year, and more than 80% of those children live in low- and middle-income countries (LMICs), where only approximately 20% of patients with cancer survive (3–8). The objective of the World Health Organization’s Global Initiative for Childhood Cancer is to achieve at least 60% survival for pediatric patients with cancer globally by 2030 (8). One strategy to help close this gap in survival between HICs and LMICs is the subspecialization and centralization of pediatric cancer care.

Subspecialization and centralization promote the pooling of expertise and resources, including therapeutic interventions, at a high-volume treatment center, which in turn increases the quality of care. Some of the many benefits of this include improved patient satisfaction, ease of providers staying current in the discipline, confidence in patient management, depth of expertise in the clinical team, efficiency in tumor boards, increased overall referrals, and increased access to clinical trials (9, 10). Many studies have shown positive results in outcomes, including survival, of patients due to subspecialization and centralization and in increasing patient volume in adult oncologic care (11–16).

Although there is paucity in literature on the centralization of pediatric oncologic care, centralization of care in cancer centers provides better outcomes and increased use of diagnostic and therapeutic procedures, compared to that in noncancer centers (17). Recent examples of successful centralization of pediatric oncology programs in HICs can be seen in The Netherlands at Princess Máxima Center for Pediatric Oncology (8, 18) and in Hungary, where the Hungarian Pediatric Oncology Network provides centralized treatment (19).

## 2 Literature search strategy

This was a non-systematic review. The Medline/PubMed literature database was searched to identify relevant articles by using multiple English terms and keywords (Sub-specialization, Centralization, Pediatric(s), Volume, Neuro-oncology, Radiology, Diagnostic Imaging, Surgery, Pathology, Oncology, Radiation Oncology, Nursing, Palliative Care, Intensive Care Unit, Physical Therapy, Occupational Therapy, etc.). No date limits were set, and only English references were used. Additionally, the review included a nonsystematic selection of relevant publications. Additional articles were then identified in the reference lists of the initial publications selected. The articles were screened by two individuals. Relevant articles were exported into Zotero software for citation management. The search continued through August 11, 2023.

## 3 Results

### 3.1 Radiology

Sub specialization and centralization promote and thrive on a multidisciplinary team approach, in which all disciplines, including radiology, communicate with each other to ensure the most accurate and highest quality care (19). An effective radiology team is complex and expensive, as it includes radiologists, nuclear medicine physicians, imaging radiographers, technologists, medical physicists, and radiochemists, among others (20). Atun R et al. (21) noted the necessity of medical imaging as a cornerstone of many oncological services. Radiology is required in the diagnosis and treatment of cancer for accurate clinical management decision making by the multidisciplinary team and optimal outcomes for the patient (20). In many cancer types, early diagnosis and proper disease staging enhance the likelihood of survival (22). Unfortunately, misdiagnoses are not uncommon, causing patients to endure unneeded, and sometimes harmful, treatment, extra costs for the families and the hospitals, and even further delay of the correct diagnosis and appropriate treatment (23).

Sub specialization and centralization can help alleviate this common dilemma in oncology by working to ensure proper diagnostic imaging and earlier diagnosis. Studies have shown major differences between initial radiologist reports and subspecialty radiologist interpretations of imaging after referral (23, 24), indicating that a second opinion by a centralized radiology team has a positive impact on accurate diagnosis and patient care. This benefit may be even greater if done earlier in the course of illness. Additionally, sub specialization and centralization of pediatric oncology radiologists can decrease hospital costs by centrally reviewing images obtained at other institutions for patients who have been referred, instead of repeating the imaging examinations (23). The Institute of Medicine (25) reported that about 25% of patients undergo unnecessary repeated tests, including imaging (26), leading to inflated costs. Establishing a centralized team of specialty-trained pediatric radiologists can reduce such unnecessary costs. In large countries, like India, Indonesia, and Ethiopia, a network of different tier centers (primary, secondary, and tertiary) can be established, and utilization of telemedicine could help to address the issue.

Sub specialization and centralization of radiology also may increase patient volume from an increase in referrals (27), leading to a more reliable supply chain for imaging diagnostics (20), a more accurate perception of epidemiology of rare tumor subtypes not usually seen in smaller centers and improved collection and storage of imaging data, which helps with long-term follow-up (8). Additionally, by establishing centralized pediatric neuro-oncology specialized radiology, survival can be improved, as seen in a study on centralized neuroradiographic review in the United States (28). In a clinical trial of patients with average-risk medulloblastoma, 17% had images that, upon review, were considered incomplete or of poor quality, and when those patients’ survival was compared with that of patients with eligible, fully assessable images, those with incomplete or poor-quality images had poorer event-free survival (28). Additionally, 10% of the patients in the study were excluded after central radiologic review, because they had disseminated or residual disease. These patients were undertreated, which significantly decreased their probability of survival (28). A subspecialized and centralized pediatric neuro-oncology radiology center also positively influences surgical outcomes (20).

### 3.2 Surgery

Many studies have shown that surgical procedures performed in high-volume centers have a higher survival rate and better outcomes, compared to those performed in low-volume centers (18, 29–36). The same has been found for individual surgeons who have performed high volumes of procedures and have more experience (29–31, 33, 35, 37). The centralization of pediatric surgical care in The Netherlands in Princess Máxima Center for Pediatric Oncology had a positive effect on both intraoperative and postoperative complications, mortality, and morbidity, when compared to pre-centralization data (18). Improvements in the care given at high-volume centers may be attributed to the skills of the staff, nursing team, and all related specialties, large number of operations, neurological intensive care unit, and high-quality advanced equipment (32). Additionally, sub specialization and centralization increase referrals, which in turn increases the experience of surgeons and hospital volume (32).

Sub specialization and high patient volume make acquiring equipment cost effective. For these reasons, surgical and radiological sub specialization and centralization should be linked because they enable hospitals to have better, more cost-effective resources (17). Another advantage of such an approach in high-volume centers is the networking among centers of excellence (32). In pediatric neuro-oncology, sub specialization and centralization of surgery offers a more aggressive approach towards many tumor resections; this is important in cancers in which survival is associated with complete resection (37, 38). One study comparing pediatric neurosurgery outcomes with surgeon experience/specialty found that the amount of residual tumor was related to the type of neurosurgeon (general, pediatric, or ASPN [American Society of Pediatric Neurosurgeons] member) who performed the procedure.

Pediatric neurosurgeons are more likely to extensively remove pediatric brain tumors than are general neurosurgeons. Additionally, lower neurological morbidity is associated with greater neurosurgical experience (e.g., ASPN members) (37). The mean number of operations performed by individual surgeons increased with sub specialization; individual general neurosurgeons perform fewer operations than do ASPN members (37).

Another study examined postoperative posterior fossa syndrome in patients with medulloblastoma; the authors found that younger age and having an operation in a low-volume center or by a low-volume surgeon increased the likelihood of posterior fossa syndrome (31). After sub specialization and centralization of pediatric brain tumor resections, mortality decreased (33). Additionally, lower rates of second brain tumor resection were associated with high surgical volumes, which can lead to lower costs in high-volume centers than in low-volume centers (34). Pediatric oncologic care (including surgery) is one of the most complex subspecialties of medical care; therefore, such cases favor centralization of care to surgical centers with high volume (34).

### 3.3 Pathology

Pathologic assessment of tissue plays a vital role in diagnosis, staging, treatment, and prognosis (39, 40), and histopathologic evaluation of tumor tissue is typically required to confirm oncologic diagnosis (39). With increasingly smaller tissue samples needed for histopathologic examination, the centralized management of those specimens helps optimize the number of examinations that can be done on the tissue sample (41). This can be achieved through specialized standard operating procedures, policies, and protocols.

One study showed that after tissue-specific protocols were implemented, quality of care increased through improved and complete pathology reporting, which led to better interpretations of pathologic findings by the clinicians (42). Pathologists play a crucial role in communicating results with clinicians (40, 41, 43). Cancer diagnosis has changed from morphology-based classification, through microscopic examinations, to a molecular-based discipline (43). Many cancers are genetically driven, and over the last few decades, targeted therapies have been developed, forcing a more specific and stratified oncologic diagnosis (43). Successful treatment depends on pathologic diagnosis (44); furthermore, better outcomes are associated with accurate stratification of treatment (42). New high-throughput technologies are becoming essential for diagnosis of oncologic pathology (43, 45, 46). Sub specialization and centralization of pathology services can be an effective way for LMICs to access new, innovative technologies.

A useful strategy for resource-limited settings is seeking a second opinion, which can lower the risk of misdiagnosis and the necessity to later amend diagnoses (44, 47, 48). The accuracy of diagnosis can be evaluated by telepathology through twinning programs; the concordance rates of accuracy are similar in both static (pictures) and dynamic (real-time video) consultations (46). Sub specialization and centralization of pathology can be implemented into LMICs through collaborations with pathologists in HICs for intensive specialty training and second opinion telepathology (49).

One study demonstrated that in low-resource settings, an intensive 6-month training specifically tailored to local needs, as opposed to full subspecialty training, provided a sufficient foundation for accurate diagnoses of pediatric cancer (46). Neuro-oncological diagnoses have very specific histologic features that can be present due to genetic, environmental, or reactive causes, among others. One study in the United States found that after a central, independent pathologic review, 28% of high-grade glioma diagnoses were reclassified as low-grade glioma (50). Those 28% of children with low-grade glioma, whose follow-up after a near-complete resection should have been monitored only, received unnecessary chemotherapy and radiation therapy, which put them at risk of acute and long-term adverse late effects. In addition, the misdiagnoses added an unnecessary extra cost burden on health care systems.

### 3.4 Neuro-oncology

Pediatric neuro-oncology is a nontechnical discipline, and the limited literature makes it difficult to quantify the impact of sub specialization and centralization of pediatric neuro-oncologists. Oncologists in LMICs typically have a heavy workload (42), with no extra time for fellowship training for sub specialization (46). Many academic hospitals in LMICs also do not have the resources to fund a full fellowship (46). An intensive 6-month specialty training was established in Brazil as a model for other low-resource settings (46). Although this model was used to train a general pathologist in pediatric cancer pathology, one can assume that it can also be used to train general oncologists to be pediatric neuro-oncologists.

The St. Jude Global Academy established the Neuro-Oncology Training Seminar as a multidisciplinary course on pediatric neuro-oncology for physicians in LMICs (51). Upon completion of the 9-week online course and the 7-day in-person workshop, physicians showed a significant improvement in comprehension of the core elements of pediatric neuro-oncology. In addition to this Academy, virtual clinical fellowship opportunities have been established through St. Jude Global and partners around the world (52). Using these novel approaches is important, because clinical fellows from LMICs who complete their medical training in HICs often decline the invitation to return to their country of origin, or they return but then leave after practicing for only a few years (53).

### 3.5 Radiation oncology

Radiation therapy is a vital element of the multidisciplinary clinical management of many pediatric cancers (54). The late adverse effects of radiation have been more clearly discerned as long-term survival of pediatric brain tumors has increased (55). Survivors of childhood brain tumors are at risk for neurocognitive, endocrine, and growth sequelae due, in part, to cranial radiation therapy (28, 54, 56). The amount and type of radiation administered are determined by the pathology, tumor location, disease progression, and age of the child (57). Large fields and high doses of radiation were used previously to treat pediatric brain tumors; however, severe late effects were seen in the first generation of survivors (55). Additionally, one study of patients with medulloblastoma showed that 71% of patients had at least one deviation in radiation, and seven patients did not receive the protocol-specified dose of radiation (58).

Because marginal deviations in radiation therapy are strongly correlated with risk of relapse (58), centralization and sub specialization are essential to ensure high quality treatment. Variations in outcome (54) indicate the need for sub specialization and centralization of radiation therapy for pediatric neuro-oncology patients; specific treatment protocols are not sufficient to positively affect quality of care without the presence of a multidisciplinary team. One important aim of radiation therapy in pediatric neuro-oncology has been to reduce the field of radiation and dose (55). Image-guided radiation therapy increases the accuracy of treatment by reducing the margin of healthy cells in the radiation beam and enabling increased dosage to the target area (54, 55). Having a specialized radiation oncologist makes this major advance possible because many technical skills are required (54).

These findings support the centralization of radiation treatment in cancer centers for an expansion of access to new, expensive resources in LMICs (59). Although 85% of the world’s population resides in LMICs, only about 33% of all radiation therapy facilities are in LMICs (60). Radiation therapy is cost-effective because of the high volume of patients a facility can treat and the long life of the equipment (21, 60). Twinning programs can also help establish radiation therapy centers and expertise in safe radiation therapy, while adapting to local and regional needs (21, 54).

### 3.6 Palliative care

Palliative care is a key component of comprehensive cancer care (59). It requires a broad, holistic, interdisciplinary approach for pain management, attending to suffering, home-based and hospice services, and bereavement (5, 10, 25, 59, 61, 62). Unfortunately, access to palliative care subspecialty is quite rare in LMICs, and if it is available, it is only at larger, more centralized locations (63). A major aspect of palliative care is to help clarify goals of treatment and care between clinicians and the patients and/or their families, which is vital throughout the illness experience (64, 65). The American Academy of Pediatrics recommends that pediatric palliative care begin at the time of the cancer diagnosis (5). Early involvement of palliative care is important for many reasons, including that children with malignant brain tumors commonly suffer from loss of their ability to communicate and present with severe symptoms at the end of life (25, 62, 66–68). Additionally, with early involvement of palliative care, advanced care planning improves, as discussions between the health care team and the patient and/or their family occur more frequently and earlier before death (69).

In patients with high-grade gliomas, palliative care consultations are associated with early change of code status to DNR (Do Not Resuscitate) and the increased use of hospice (70). Additionally, early integration promotes the continuation of relationships between the multidisciplinary team (including palliative care specialists) and the family, while avoiding the transfer of care or the introduction of new palliative care specialists during the last days of a child’s life (67). Palliative care consultations are associated with a shorter duration of stay in the hospital without any effect on mortality, and fewer deaths in the intensive care unit (64, 69). Integration of palliative care can also improve the quality of life by using less-aggressive treatment at the end of life and can improve survival (71). Interestingly, after the integration of palliative care, the number of hospital admissions at the end of life may be reduced (69); this was especially true for children with brain tumors (72). One study in Italy found that after the integration of palliative care, none of the patients with brain tumors had uncontrolled pain; almost 60% of patients received palliative sedation, and 44% died at home (66).

Pediatric palliative care includes patients and parents in clinical decision making; the wishes of the patient and/or parents are considered (65). Parents feel more comfortable discussing death with their child after consultations with the palliative care team (61). From the parents’ point of view, clear and honest communication from the doctor about their child’s prognosis is one of the strongest determinants of high-quality physician care (73). For parents of children with cancer, preparedness may be their only sense of control over unpredictable and devastating circumstances (73). Integrating palliative care aids in the grief and bereavement journey of surviving family members (68).

### 3.7 Supportive therapies

As the incidence and survival of pediatric brain tumors are increasing, supportive therapies, such as physical therapy, occupational therapy, speech therapy, neuropsychology, dental service, optometry, and audiometry, continue to be essential (74–77). Treatments for brain tumors can cause detrimental sequelae, including neurocognitive deficits, neurologic complications, endocrine abnormalities, gastrointestinal impairments, eye-related complications, and hearing loss (56, 74, 76, 78–81). For example, posterior fossa syndrome arises postoperatively in 40% of children with brain tumors located in the posterior fossa and presenting symptoms of impaired communication (often mutism), depressed mood, and loss of motor skills (82). Neuropsychological evaluations and interventions improve outcomes in pediatric neuro-oncology patients (56, 57, 83). Incorporating inpatient rehabilitation for patients with neurological disabilities due to brain tumors can also lead to functional improvement in activities of daily living (79, 84). Rehabilitation specialists in multiple disciplines (e.g., physical therapy, occupational therapy, and speech therapy) are required to optimize overall patient function (74). Early identification of these sequelae is vital to optimize normal speech, communication, language development, and social interactions for improved quality of life (85).

The Rehabilitation Medicine Department of the Clinical Center at the National Institutes of Health suggests integrating rehabilitation care from diagnosis and throughout the patient’s journey (86). The oncology team must screen patients for levels of function to ensure accurate and timely referral during the course of treatment and long-term follow-up (74, 77). Including rehabilitation providers on the multidisciplinary teams/rounds supports patients and families by ensuring consistent care providers throughout treatment (75), coordinated scheduling of rehabilitation services and anticancer treatment (74, 87), and improved discharge planning that can reduce the likelihood of re-admission to acute care (75, 87) and promote successful school transitions (88) (**Table 1**).

**Table 1:** Depicts the key findings of every discipline and benefits of sub specialization and centralization of pediatric neuro-oncology services.

Discipline	Key findings	Benefits
Radiology	Misdiagnosis common; second opinion improves accuracy; centralized subspecialty review reduces repeat imaging and costs; telemedicine helps in large LMICs	Accurate diagnosis, early detection, cost reduction, improved survival outcomes
Surgery	High-volume centers and experienced surgeons improve survival; fewer complications; better resection rates; MRI-guided resections improve extent of resection; linked with reduced mortality post-centralization	Better outcomes, fewer reoperations, cost effective equipment use, improved surgical expertise
Pathology	Accurate histopathology essential; misclassification common (28% in gliomas); second opinions reduce errors; molecular diagnostics emerging; telepathology valuable for LMICs	Accurate diagnosis, prevents overtreatment, improves prognosis estimation, access to advanced testing
Neuro-Oncology	Limited specialists in LMICs; heavy workload hinders training; short-term specialty training effective; online and virtual fellowship programs successful	Capacity building, improved disease management, retention of trained specialists
Radiation Oncology	High deviations from protocol (71% in one study); relapse risk increases with radiation deviations; advanced techniques like IGRT improve precision; equipment scarce in LMICs (33% availability)	Improved survival, reduced toxicity, access to advanced technology via centralized centers, twinning programs support LMICs
Palliative Care	Limited access in LMICs; early involvement improves symptom control, communication, and family support; reduces hospital deaths and ICU stays; improves quality of life	Holistic care, better end-of-life outcomes, reduced aggressive futile care, improved family satisfaction
Supportive Therapies	Essential for managing neurocognitive, motor, speech, endocrine and psychological sequelae; early rehabilitation improves long-term function; multidisciplinary rehab reduces readmissions	Better functional outcomes, improved quality of life, coordinated long-term care

## 4 Discussion

Sub specialization and centralization have been used as a strategy to reduce the disparity in survival of children with cancer in LMICs versus those in HICs and has led to increased volume, development of molecular diagnostics, innovative treatments, and outcomes comparable to those in HICs (89). Sub specialization and centralization of rare, complex, and multidisciplinary specialties, like pediatric neuro-oncology, will help increase the quality of care and patient outcome. LMICs that have established pediatric neuro-oncology programs include Malaysia (51), Jordan (89), and Pakistan (90). After the establishment of these programs, survival and patient volume significantly increased, in terms of steep increase in referrals, better tumor board participation, and providing best-possible available treatment options in resource-constrained settings. Sub-specialized and centralized pediatric neuroradiology, surgery, pathology, oncology, radiation oncology, nursing, palliative care, intensive care, and rehabilitation services are all necessary aspects of multidisciplinary care in pediatric neuro-oncology. The concept of decentralization is appealing for LMIC governments; however, for rare diseases like pediatric brain tumors, sub specialization and centralization of services deceases the chances of relapse by providing good staging (28), correct diagnosis (50), and proper therapies upfront (18). It also decreases morbidity and complications (31, 37) and avoids unnecessary therapies (50), which translates to less short- and long-term costs. Governments and tertiary centers should be sensitive to local needs and the burden of travel, losing working time, and housing, so centralization does not become a burden on patients and families. In LMIC, a hub and spoke model connects resource-rich central facilities like tertiary cancer care hospitals (hubs) with peripheral facilities like primary and secondary care hospitals (spokes) to improve healthcare delivery. The hub provides technical expertise, training, and materials, while the spokes offer basic services, creating a system where specialized care can reach underserved areas through methods like emergency services and elective patient transfers. This model is well-suited for areas like cancer care and acute coronary syndromes to ensure best outcomes.

## 5 Conclusions

Caring for pediatric neuro-oncology patients is complex and requires a multidisciplinary team of radiologists, surgeons, pathologists, oncologists, radiation oncologists, nurses, palliative care specialists, intensivists, and therapists, among others. Sub specialization and centralization promote high-quality communication and collaboration among multidisciplinary care providers. In low-resource settings and many LMICs, sub specialization and centralization are an effective use of resources to improve quality of care for the many patients in areas where they previously did not have access to multidisciplinary care, thereby ensuring early diagnosis and effective treatment. Additionally, twinning programs and telemedicine are an effective way to help establish pediatric neuro-oncology programs in low-resource settings. Effective twinning and other models continue to close the gap in survival of children with brain tumors in HICs and those in LMICs. Although sub specialization and centralization have been successful, barriers to centralization may include the distance to travel and financial burden associated with that travel, which could lead to underserved care. Thus, housing, transportation, and financial support for patients should be considered when establishing a centralized pediatric neuro-oncology service in low-resource settings. More prospective research in this area is needed to determine the true impact of sub specialization and centralization on quality of care, survival, and psychosocial outcomes in pediatric neuro-oncology. Smaller LMICs could follow Netherlands steps in its novel approach to childhood cancers–one pediatric cancer hospital for the whole country while for larger LMIC model like hub and spoke could be beneficial where there is a regional center for excellence which could be the hub.

## 6 Limitations

As the review is non-systematic, the limitations include the lack of a standardized, transparent, and comprehensive methodology, leading to potential bias, inconclusive results, and an inability to fully assess the quality of the included studies.
